# Gastric Adenocarcinoma in a Pregnant Woman at 33 Weeks of Gestation: A Case Report of Krukenberg Tumor Complicated by Uterine Rupture

**DOI:** 10.7759/cureus.91488

**Published:** 2025-09-02

**Authors:** Omar Lotfi, Reem Elnemr, Mohsen Mosallam, Neha Uddin, Marwa Hassan

**Affiliations:** 1 Obstetrics and Gynecology, The Grange University Hospital, Cwmbran, GBR; 2 Obstetrics and Gynecology, Zagazig University Hospitals, Zagazig, EGY; 3 Respiratory Medicine, Blackpool Victoria Hospital, Blackpool, GBR; 4 Anesthesiology and Critical Care, Zagazig University Hospitals, Zagazig, EGY

**Keywords:** chemotherapy, gastric cancer, krukenberg tumor, multidisciplinary management, pregnancy

## Abstract

A 34-year-old woman, gravida 2 para 1, presented at 33 weeks of gestation with significant weight loss, anorexia, abdominal pain, and a single episode of hematemesis. On examination, she appeared cachectic, with generalized abdominal tenderness. Imaging revealed bilateral adnexal masses and ascites, and endoscopy identified a gastric mass. Biopsy confirmed signet-ring cell adenocarcinoma. Laboratory workup showed elevated cancer antigen 125 and hypoalbuminemia.

Plans were made to deliver at 37 weeks to maximize fetal maturity. However, at 35 weeks, she developed acute abdominal pain and fetal bradycardia. Emergency laparotomy revealed uterine rupture. A total hysterectomy with bilateral salpingo-oophorectomy was performed. The infant, delivered with no signs of life, died four days later despite supportive care.

Histopathology confirmed Krukenberg tumors (bilateral ovarian metastases) and peritoneal spread. Postpartum positron-emission tomography/computed tomography revealed osseous metastases. The patient commenced treatment with folinic acid, fluorouracil, and oxaliplatin version 6 and nivolumab, later receiving palliative radiotherapy and transitioning to paclitaxel.

This case captures the diagnostic and therapeutic challenges of managing advanced gastrointestinal cancer in late pregnancy. It also highlights the emotional and clinical weight of navigating maternal care amid rapidly evolving disease and obstetric emergencies. Multidisciplinary coordination was critical throughout her journey.

## Introduction

Gastric cancer during pregnancy is exceptionally rare, with an estimated incidence of just one in 1,000,000 pregnancies [1]. Its diagnosis is often delayed, as hallmark symptoms such as nausea, vomiting, and weight loss are easily mistaken for normal pregnancy-related changes. This overlap in symptomatology frequently leads to advanced-stage detection, contributing to poor maternal and fetal outcomes.

Among the most serious complications is the development of a Krukenberg tumor, a metastatic ovarian tumor originating from a primary gastrointestinal adenocarcinoma, most commonly of gastric origin [2]. These tumors are typically bilateral and aggressive, carrying a poor prognosis, with a median survival reported as only a few months in pregnant patients. In pregnancy, they pose additional challenges: their rapid progression and nonspecific abdominal or pelvic symptoms can mimic physiological gestational changes. At the same time, imaging and therapeutic options are limited by concerns for fetal safety [3-5].

This case highlights the diagnostic and therapeutic challenges of managing advanced gastric malignancy in pregnancy. It underscores the critical need for early recognition, high clinical suspicion, and a multidisciplinary approach to guide individualized treatment plans that optimize outcomes for both mother and child.

## Case presentation

A 35-year-old woman, gravida 2 para 1+0, presented to the emergency department at 33 weeks of gestation with progressive gastrointestinal and constitutional symptoms. She reported a 15 kg unintentional weight loss from her prepregnancy baseline, persistent anorexia, vague abdominal discomfort, and a single episode of hematemesis. Her antenatal course had otherwise been unremarkable until the onset of these symptoms in the third trimester.

On examination, her body mass index was 23 kg/m². She appeared cachectic, but her vital signs were within normal limits. Abdominal examination revealed distension and generalized tenderness without signs of peritonism. Baseline laboratory investigations were obtained to assess nutritional status, tumor markers, and organ function; the results of which are summarized in Table 1.

**Table 1:** Laboratory findings CA: cancer antigen; LDH: lactate dehydrogenase; INR: international normalized ratio; PTT: partial thromboplastin time; ALT: alanine aminotransferase; AST: aspartate transaminase; G.O.T: glutamic oxaloacetic transaminase; G.P.T: glutamic-pyruvic transaminase; WBCs: white blood cells; NEUT: neutrophils; MONO: monocytes; BASO: basophils; LYM: lymphocytes; RBCs: red blood cells; PCV: packed cell volume; MCV: mean corpuscular volume; MCH: mean corpuscular hemoglobin; MCHC: mean corpuscular hemoglobin concentration; RDW: red cell distribution width; PDW: platelet distribution width; MPV: mean platelet volume

Test	Result	Unit	Reference range
CA 125	5.7 → 869	U/mL	1.9-16.3
CA 15-3	9.71	U/mL	0-30
Albumin	2.46	g/dL	3.5-5.2
Total protein	5.47	g/dL	6.4-8.3
Sodium (Na)	131	mmol/L	136-145
Potassium (K)	3.46	mmol/L	3.5-5.1
Calcium	7.89	mg/dL	8.6-10
Phosphorus	2.46	mg/dL	2.5-4.5
Magnesium	1.67	mg/dL	1.6-2.6
LDH	319	U/L	135-225
Fibrinogen	7.1	g/L	1.8-3.6
Prothrombin time	10.4	seconds	11-14
Prothrombin concentration	125.9	%	80-120
INR	0.91	-	0.8-1.2
PTT	33.3	seconds	26-42
Total bilirubin	0.77	mg/dL	Up to 1.2
Direct bilirubin	0.54	mg/dL	Up to 0.2
ALT (G.P.T)	11	U/L	Up to 32
AST (G.O.T)	32.7	U/L	Up to 32
Serum creatinine	0.57	mg/dL	0.5-0.9
Serum urea nitrogen	5.4	mg/dL	6-20
WBCs	10.6	×10^3^/uL	4-11
NEUT	8.5	×10^3^/uL	2-7
MONO	0.3	×10^3^/uL	0.2-1
BASO	0.3	×10^3^/uL	Up to 0.4
LYM	1.5	×10^3^/uL	4-5.2
RBCs	4.2	×10^6^/uL	4.6-6
Hematocrit (PCV)	34.8	%	36-45
Hemoglobin (Hb)	11.9	g/dL	11.5-15.5
MCV	83.1	fL	80-100
MCH	28.4	pg	27-33
MCHC	34.2	%	31-37
RDW	16.8	%	-
Platelet count	331	×10^3^/uL	150-450
PDW	8.3	%	10-20
MPV	8.4	fL	7.1-11.2

**Figure 1:**
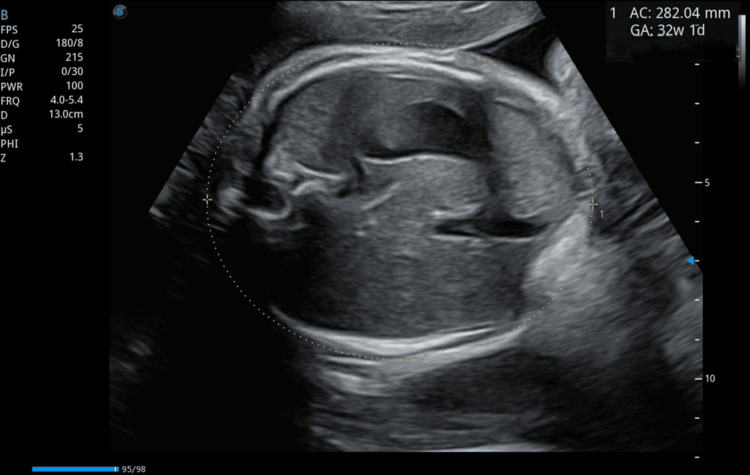
Ultrasound image demonstrating a transverse section of the fetal abdomen at 32 weeks and one day of gestation

**Figure 2:**
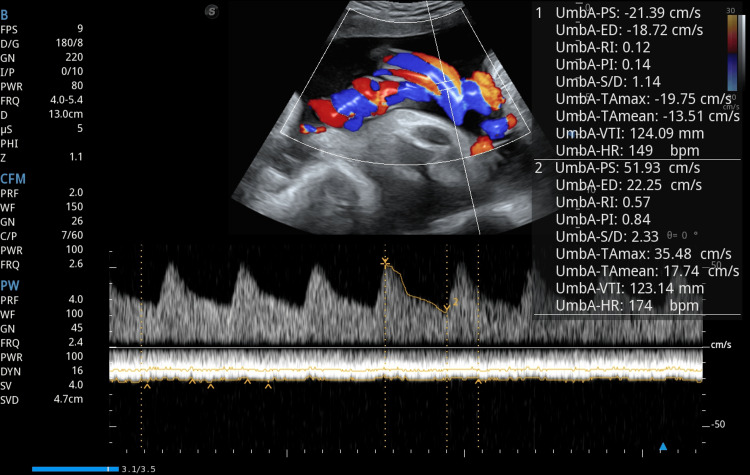
Umbilical artery Doppler ultrasound demonstrating waveforms and blood flow parameters in a fetus at 32 weeks of gestation

**Figure 3:**
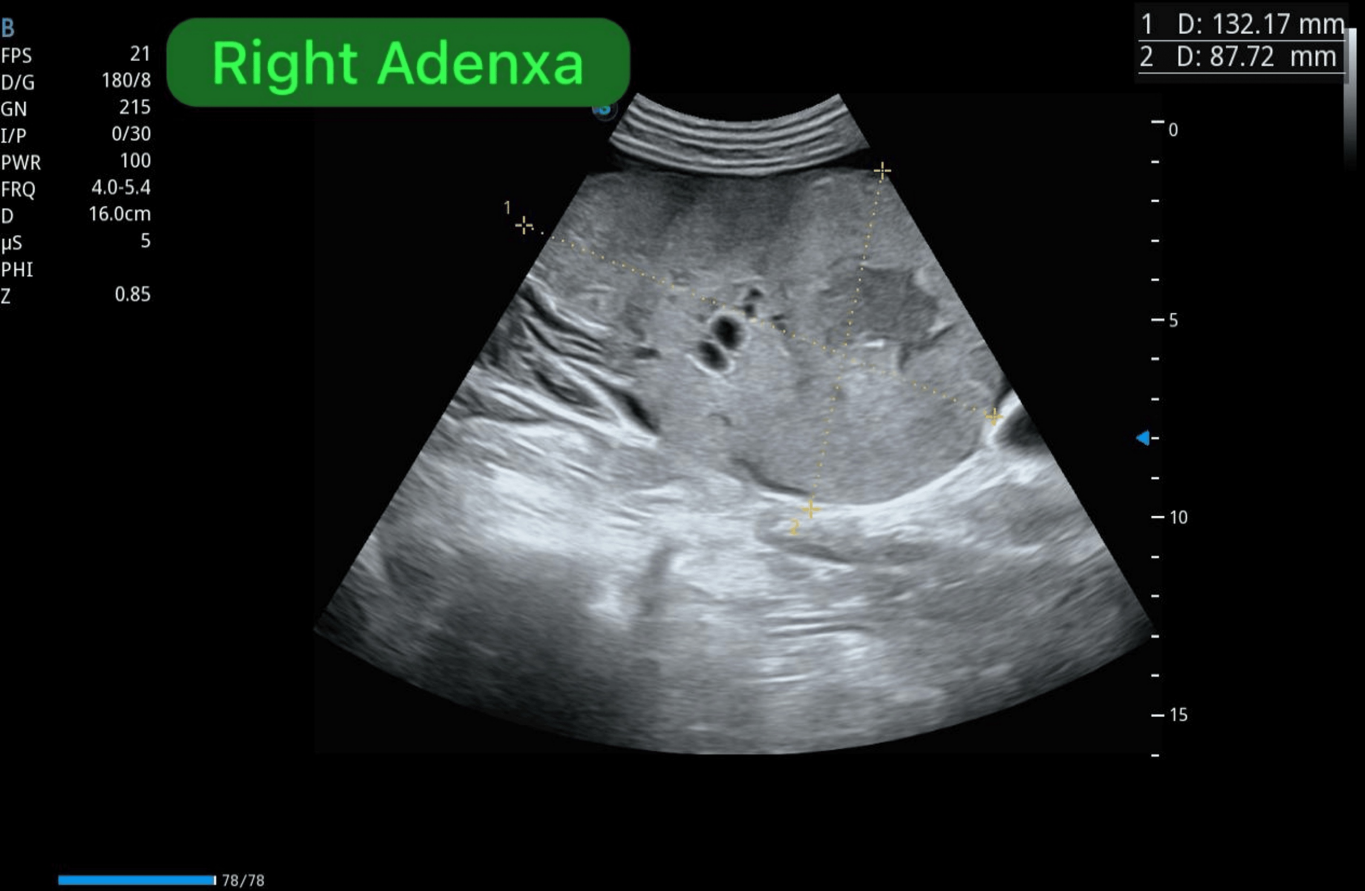
Transabdominal ultrasound of the right adnexa demonstrating a large complex adnexal mass

**Figure 4:**
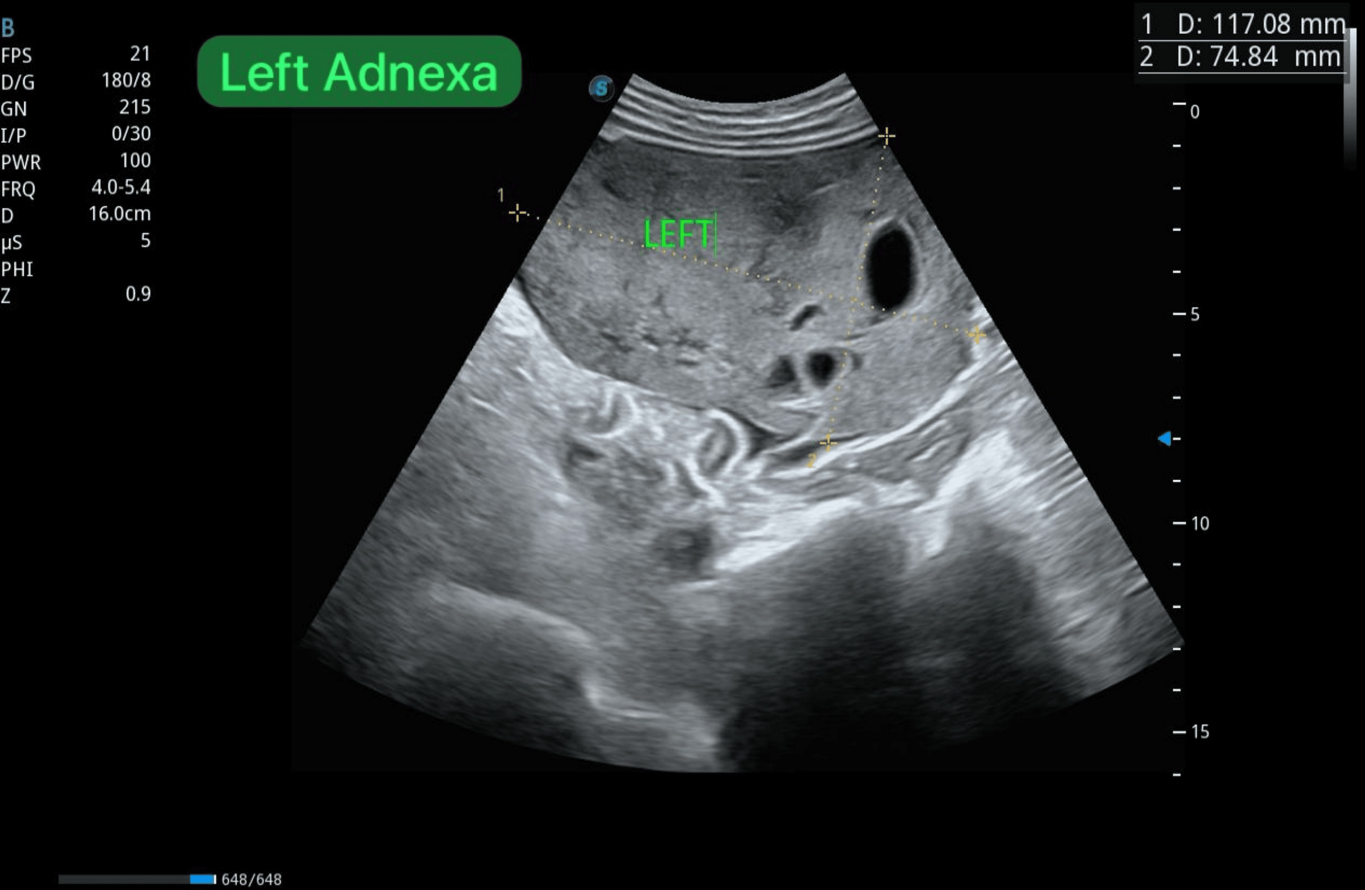
Transabdominal ultrasound of the left adnexa revealing a large complex adnexal mass

Obstetric ultrasound confirmed a single viable fetus corresponding to 32 weeks and one day of gestation, with normal umbilical artery Doppler indices, indicating adequate placental perfusion at the time of assessment (Figures 1, 2). Notably, the scan also identified large bilateral adnexal masses, measuring 132 × 87 mm on the right and 117 × 74 mm on the left (Figures 3, 4). Both masses appeared solid with moderate vascularity and were associated with moderate ascites, raising the suspicion of bilateral ovarian neoplasms.

**Figure 5:**
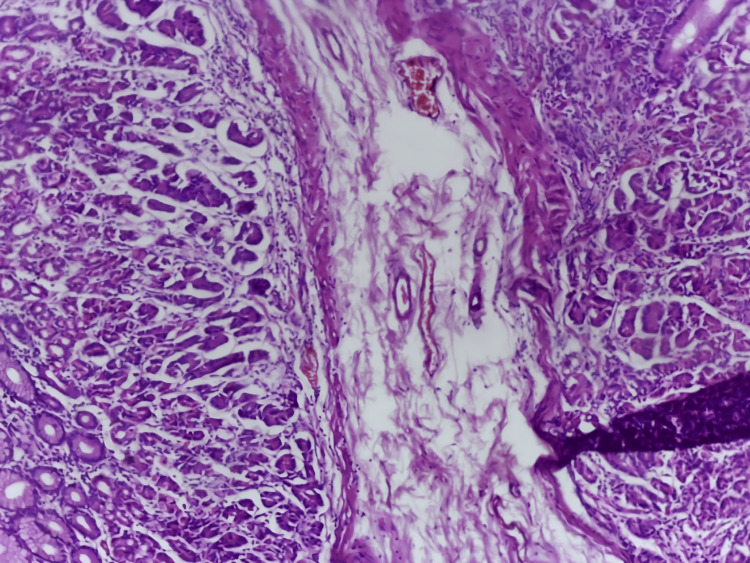
Histopathological section of gastric mucosa demonstrating poorly cohesive adenocarcinoma with stromal infiltration (H&E stain, high-power view) H&E: hematoxylin and eosin

Given her gastrointestinal symptoms, cachexia, and the concerning imaging findings, a gastrointestinal malignancy was suspected. A gastroenterology consultation was obtained, and esophagogastroduodenoscopy revealed a gastric mass. Histopathological analysis of biopsy samples confirmed poorly cohesive infiltrating adenocarcinoma (Figure 5) with signet-ring cells, consistent with advanced gastric cancer.

A multidisciplinary team involving oncology, maternal-fetal medicine, and surgical specialties decided to defer radical surgical intervention in favor of continuing the pregnancy to term, provided the mother remained stable. The goal was to balance fetal viability with maternal prognosis.

**Figure 6:**
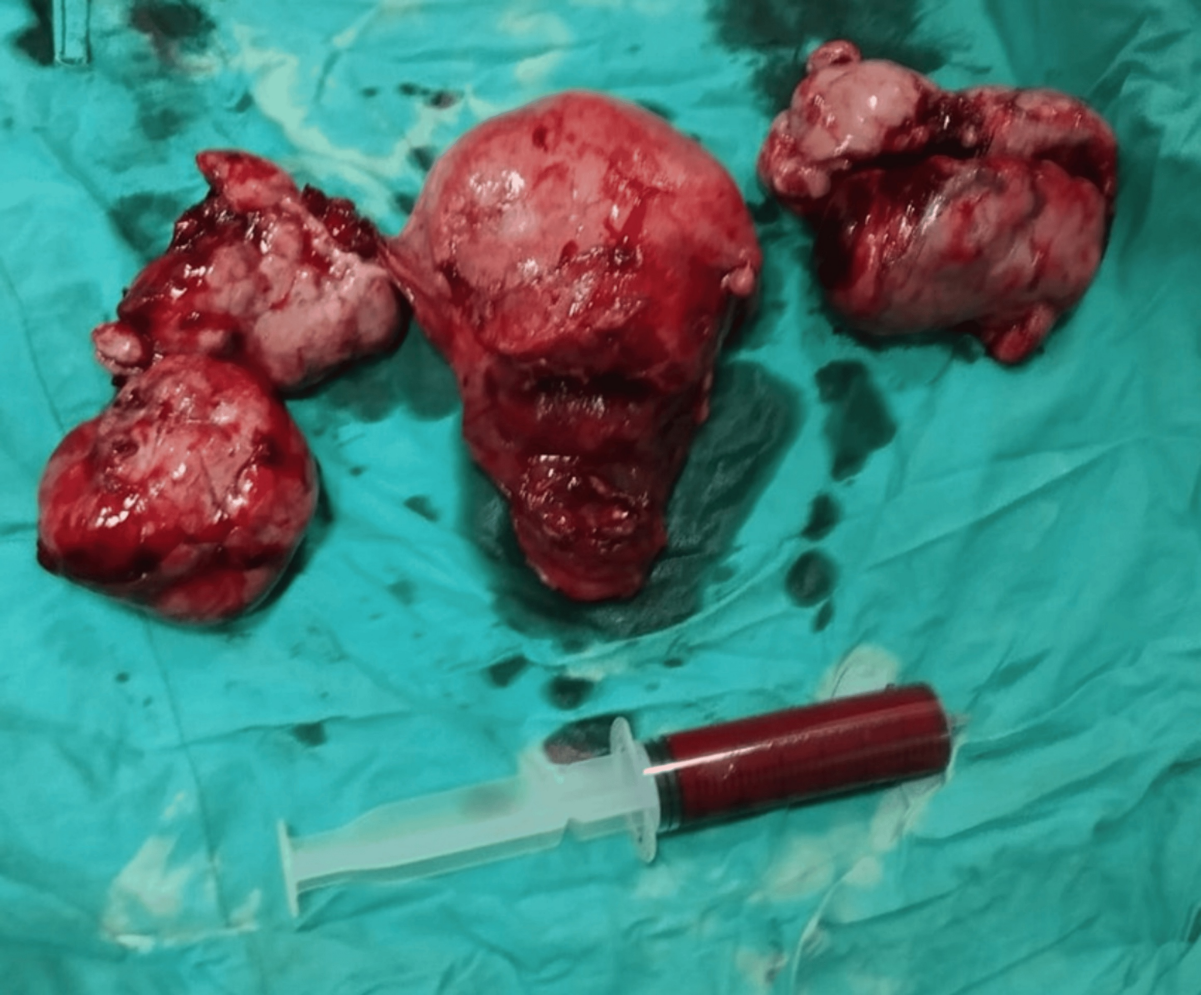
Gross surgical specimen following total hysterectomy with bilateral salpingo-oophorectomy and peritoneal fluid aspiration

At 35 weeks of gestation, the patient experienced sudden-onset severe abdominal pain accompanied by fetal bradycardia. Emergency laparotomy was performed, which revealed uterine rupture. A total hysterectomy with bilateral salpingo-oophorectomy was performed (Figure 6). The neonate was delivered via classical cesarean section and required immediate mechanical ventilation. Despite supportive care, the neonate died on the fourth postnatal day.

**Figure 7:**
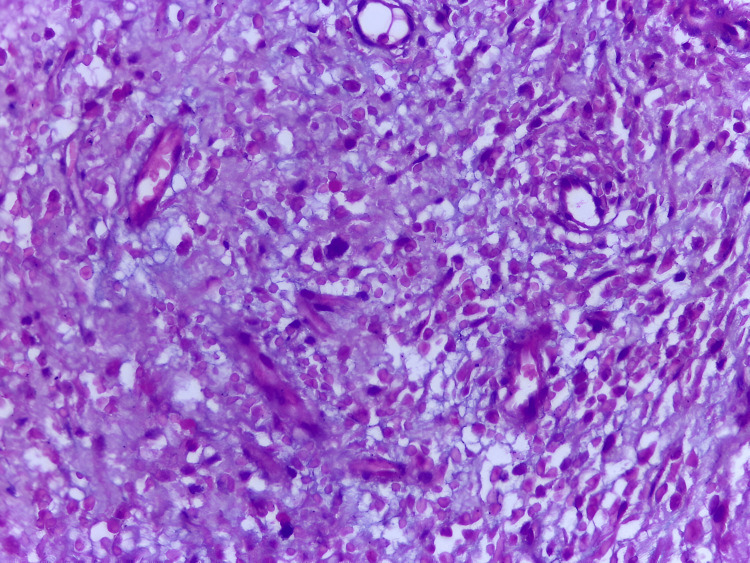
Histopathologic image showing signet-ring cell carcinoma with abundant mucin displacing the nuclei peripherally (H&E stain, high-power field) H&E: hematoxylin and eosin

During the operation, peritoneal fluid and omental biopsies were collected. Intraoperative oncology consultation recommended delaying gastric resection until further metastatic evaluation and maternal stabilization were achieved. Histopathological analysis confirmed bilateral ovarian metastases consistent with Krukenberg tumors, and peritoneal cytology revealed malignant cells (Figure 7).

**Figure 8:**
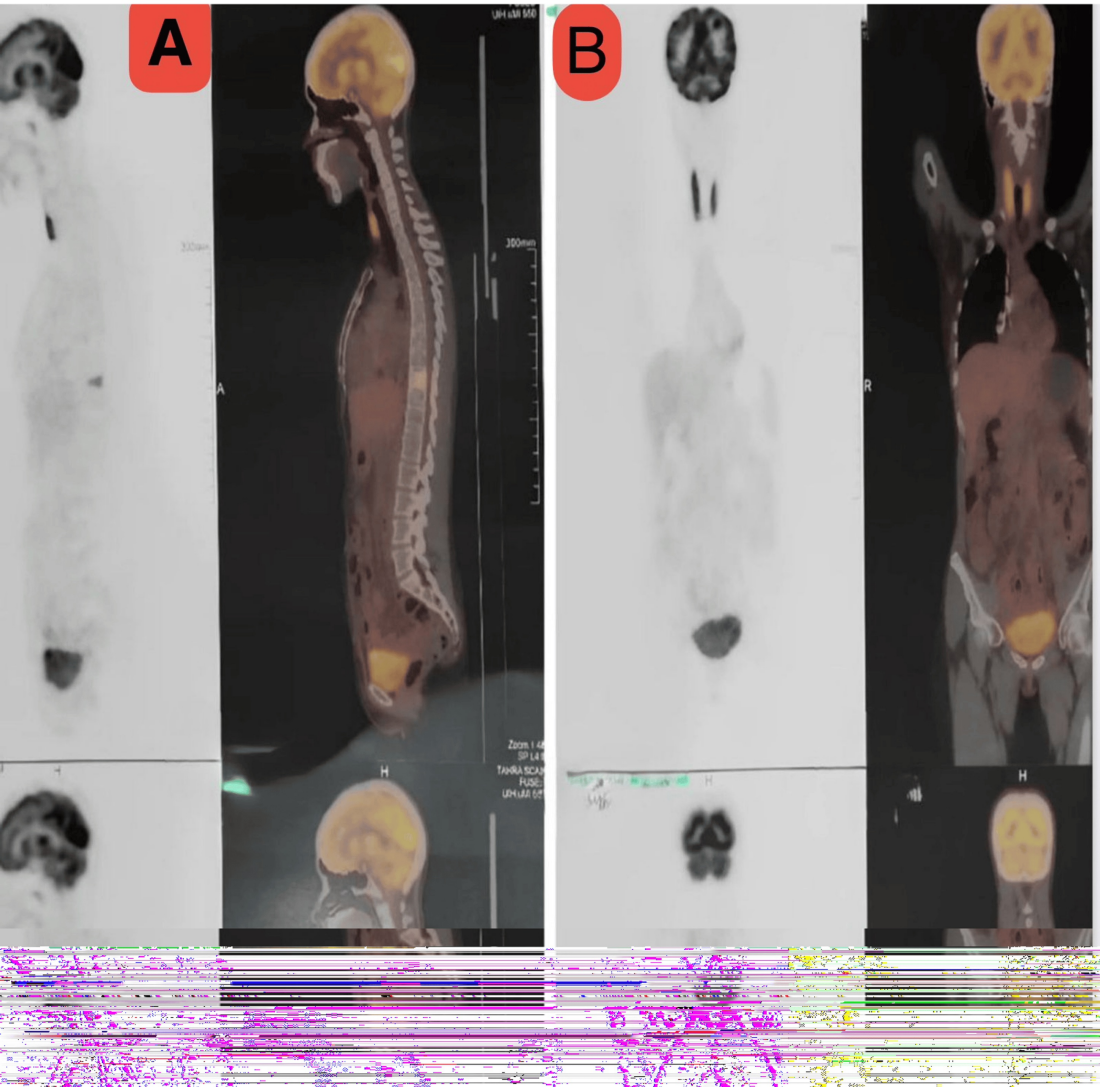
(A) Sagittal fused PET/CT images showing osseous metastases in the thoracic spine. (B) Coronal fused PET/CT images demonstrating peritoneal and osseous metastases PET/CT: positron-emission tomography/computed tomography

Subsequent positron-emission tomography/computed tomography (PET/CT) imaging showed progressive thickening of the gastric wall (SUVmax 2.4) and new osseous metastases involving the thoracic spine (T10) and sacrum (SUVmax 3.5). Ascites was mild and stable, and no visceral or nodal progression was observed (Figure 8).

The patient began systemic chemotherapy with folinic acid, fluorouracil, and oxaliplatin version 6 (FOLFOX-6) administered biweekly, in combination with intravenous nivolumab (240 mg). Despite treatment, follow-up imaging demonstrated further disease progression, particularly in the osseous sites. She subsequently received palliative radiotherapy to the spine and sacrum (8 Gy in a single fraction) for pain relief. Due to continued progression, the patient was transitioned to second-line chemotherapy with paclitaxel.

## Discussion

Gastric cancer during pregnancy is exceedingly rare and is frequently diagnosed at an advanced stage due to the substantial overlap between its presenting symptoms, such as nausea, vomiting, abdominal pain, and weight loss, and the common physiological changes of pregnancy [5]. This diagnostic challenge often results in delays that significantly impact maternal and fetal outcomes. The most prevalent histologic subtype associated with pregnancy-related gastric cancer is poorly cohesive adenocarcinoma, particularly signet-ring cell carcinoma, which is known for its aggressive behavior and early peritoneal and ovarian dissemination, commonly manifesting as Krukenberg tumors [6,7].

Krukenberg tumors are typically characterized by bilateral ovarian involvement, high vascularity on imaging, and the presence of signet-ring cells on histological examination. Peritoneal carcinomatosis accompanies these tumors in a significant proportion of cases, up to 76%, making it a helpful distinguishing feature from primary ovarian malignancies [2,8]. When interpreted in combination with serum tumor markers and advanced imaging, these features can guide accurate diagnosis and staging.

In the present case, the patient presented during the third trimester with persistent anorexia, progressive weight loss, and hematemesis. Initially, her symptoms were considered benign and attributed to gestational changes, which contributed to a delay in diagnostic evaluation. However, when symptoms persisted and her clinical condition deteriorated, further investigation was undertaken. Upper gastrointestinal endoscopy ultimately revealed a gastric mass, and histopathology confirmed the diagnosis of signet-ring cell carcinoma. Endoscopy remains the gold standard for diagnosing upper gastrointestinal malignancies and is considered safe during pregnancy when performed with appropriate precautions, particularly in the second trimester [9].

Serum cancer antigen (CA) 125, although a nonspecific marker, can support the assessment of ovarian involvement, especially when levels are markedly elevated. In this patient, CA 125 rose from 5.7 to 869 U/mL over a short interval, reflecting the burden of disease. When interpreted alongside imaging findings and validated predictive tools such as the ADNEX model, CA 125 can be instrumental in differentiating primary ovarian cancer from metastatic lesions [10].

Several case reports have documented gastric cancer with Krukenberg tumor in pregnancy, often associated with poor prognosis due to the tumor's aggressive nature and the advanced stage at diagnosis. The median overall survival for patients with Krukenberg tumors of gastric origin is approximately 11 months. Fetal outcomes are also adversely affected, as maternal disease frequently necessitates early delivery to manage complications. This urgency increases the risk of fetal morbidity and mortality. In some cases, preeclampsia has been observed in conjunction with Krukenberg tumors during pregnancy, requiring premature delivery and contributing to poor neonatal outcomes [11].

Management of gastric cancer in pregnancy demands a delicate balance between optimizing maternal prognosis and ensuring fetal viability. Contemporary recommendations stress the importance of prompt endoscopic evaluation in pregnant patients presenting with alarm symptoms, regardless of gestational age [12]. Surgical decision-making should incorporate gestational age, tumor staging, and maternal reproductive goals [12]. Chemotherapy may be administered after the first trimester, although it is typically reserved for palliative intent in metastatic disease [13]. Radiotherapy, when necessary, is generally deferred until the postpartum period and is primarily used for palliation of symptomatic metastases.

In this case, surgery was initially deferred in accordance with the patient’s wishes after thorough multidisciplinary counseling. At the time of diagnosis, earlier surgical intervention was offered but would have required hysterectomy with bilateral salpingo-oophorectomy and permanent loss of fertility. The patient therefore elected to continue the pregnancy with the goal of reaching 37 weeks to maximize fetal maturity. Unfortunately, at 35 weeks, she developed acute abdominal pain and fetal bradycardia. Emergency laparotomy revealed uterine rupture, necessitating a total hysterectomy with bilateral salpingo-oophorectomy. Histological examination confirmed bilateral Krukenberg tumors. Although the neonate was delivered alive, he died after four days despite intensive care. This outcome illustrates the precarious balance between maternal prognosis and fetal viability, as efforts to optimize neonatal maturity exposed the patient to significant maternal risk. In retrospect, earlier surgical intervention might have reduced the likelihood of uterine rupture but at the expense of fetal survival, underscoring the importance of transparent counseling, respect for patient autonomy, and individualized multidisciplinary decision-making in managing such complex cases [14].

The mechanism underlying the uterine rupture in this patient is uncertain but may be multifactorial. Malignant infiltration of the myometrium by metastatic disease could have weakened the uterine wall, predisposing it to rupture. Mechanical stress from the rapidly enlarging gravid uterus at 35 weeks, compounded by peritoneal carcinomatosis and adnexal masses, may also have contributed to increased intra-abdominal pressure and wall tension. Furthermore, rapid disease progression itself has been linked to spontaneous uterine rupture in rare oncologic cases, even in previously unscarred uteri [15]. Similar cases have been reported in the literature, where malignancy-associated uterine rupture occurred in the absence of prior surgery, supporting the role of tumor infiltration and disease burden as critical contributing factors [15]. These factors likely acted synergistically in this patient, resulting in this catastrophic event.

Postoperatively, PET/CT imaging confirmed stage IV disease, including new osseous metastases in the thoracic spine and sacrum. The patient was initiated on FOLFOX-6 chemotherapy, combined with nivolumab (Opdivo, Bristol-Myers Squibb, Princeton, NJ). However, further disease progression prompted the administration of palliative radiotherapy to the spinal lesions (8 Gy in a single fraction), followed by a second-line regimen of pembrolizumab (Keytruda, Merck & Co., Rahway, NJ) and paclitaxel (Taxol, Bristol-Myers Squibb).

This case underscores the diagnostic challenges of malignancy during pregnancy and the significant consequences of delayed recognition. It also highlights the importance of a multidisciplinary approach when navigating complex decisions that affect both maternal and fetal outcomes. Early suspicion and prompt evaluation are critical in ensuring timely diagnosis and in implementing interventions that offer the best possible balance between therapeutic efficacy and obstetric safety.

## Conclusions

This case highlights the diagnostic complexity of malignancy during pregnancy and underscores the need for a high index of clinical suspicion in patients presenting with persistent or atypical gastrointestinal symptoms. Early endoscopic evaluation should not be delayed when red flag symptoms are present. Multidisciplinary coordination and individualized treatment planning remain essential to optimize maternal care while considering fetal viability. As evidence remains limited, further research and consensus guidelines are necessary to support timely diagnosis and appropriate management of cancer in pregnancy.
